# Supplementary material for: Transcriptome analysis of cortical tissue reveals shared sets of downregulated genes in autism and schizophrenia
Source: Transl Psychiatry. 2016 May 24;6(5):e817–. doi: 10.1038/tp.2016.87 (PMC5070061; doi:10.1038/tp.2016.87)
Supplement: Supplementary Information [file tp201687x1.docx]

**SUPPLEMENTAL DATA TABLE OF CONTENTS**

**Supplemental Methods** – Details on Methods Included in Manuscript

**Detailed Methods: Pathway Analysis of DCGs**

**Detailed Methods: Enrichment for Genetic Signal Analysis**

**Supplemental Discussion** -- Details on Methods Included in Manuscript

**Accounting for Unknown Covariates Is Critical in Transcriptome Analyses**

**Supplemental Figures**

Supplemental Figure 1 : Differential Gene Expression Across AUT, SCZ, and BPD

Supplemental Figure 2 : Correlation of Genes Differentially Expressed Across All Three Disorders

Supplemental Figure 3 : Assessing the significance for Correlations of Cross-Disorder Transcriptomic Similarity

Supplemental Figure 4 : Accounting for Unknown Covariates Affects Correlation

Supplemental Figure 5 : GO Analysis of DCEGs

Supplemental Figure 6 : GO Analysis of cross-disorder DEGs

Supplemental Figure 7 : GO Analysis of DCEGs

Supplemental Figure 8 : Enrichment of DEGs among GWAS signal

Supplemental Figure 9 : Enrichment of DEGs among GWAS at a more permissive p-value cutoff (p<0.1)

Supplemental Figure 10 : Enrichment of DEGs among GWAS signal at a more stringent p-value cutoff (p<0.01)

Supplemental Figure 11 : Enrichment of DEGs among all genes (no gene based GWAS p-value cutoff imposed)

**Supplemental Tables**

Supplemental Table 1 : Covariates from AUT data set

Supplemental Table 2 : Covariates from the SMRI data set

Supplemental Table 3 : Differential Gene Expression Analysis (Z-Scores)

Supplemental Table 4 : Cross-Disorder DGEA Z-Scores

Supplemental Table 5 : DAVID Pathway Analysis for cross-disorder DEGs controlling for # of genes input

Supplemental Table 6 : MSigDB GO Analysis Results

Supplemental Table 7 : Association Signal Enrichment Results across multiple p-value cutoffs
