# Supplementary material for: Transcriptome analysis of cortical tissue reveals shared sets of downregulated genes in autism and schizophrenia
Source: Transl Psychiatry. 2016 May 24;6(5):e817–. doi: 10.1038/tp.2016.87 (PMC5070061; doi:10.1038/tp.2016.87)
Supplement: Supplementary Information [file tp201687x2.docx]

**Supplemental Methods**

**Detailed Methods: Pathway Analysis of DCGs**

Cross-disorder Z-scores were calculated, such that:

Z_AUT-SCZ_ = (Z_AUT_ + Z_SCZ_)/sqrt(*k*)

where *k* is the number of comparisons made (here, *k*=2). A one-sided t-test was used to compare Z-scores between genes in the pathway and genes not in the pathway. To assess significance for each pathway in each of the cross-disorder comparisons, the absolute value of cross-disorder Z-scores for those genes in the pathway were compared to the absolute value of cross-disorder Z-scores for those genes not in the pathway using a one-sided t-test under the alternative hypothesis that Z-scores in the pathway were enriched for significant Z-scores relative to the genes not in the pathway. The 1,285 GO categories for which we had gene expression data for at least five genes in the pathway were included for analysis.

**Detailed Methods: Enrichment for Genetic Signal Analysis**

The following tests were conducted allowing for up to one million permutations: ‘logistic-minsnp-gene-perm’ and ‘logistic-gwis-perm’. LD was calculated on the fly using LD computed from HapMap Phase 1 CEU imputation data. Default settings were used, aside from the following: the flank parameter (region on either side of the gene for investigation) was set to 15kb, phenotype variance was estimated at 0.01, and a maf-cutoff of 0.01 was used. Sample sizes were estimated to be approximately 1.5x the number of cases used in each individual analysis. Accordingly, the sample sizes used as input were 10,000, 15,000 and 20,000 for AUT, BPD, and SCZ respectively. Downstream gene-based p-values were compiled such that the GWiS^27^ p-value was used for all genes assigned p-values not equal to one (signifying that no permutations were carried out in GWiS). Otherwise, the more permissive minSNP-P p-value was assigned to the gene. The minSNP-P simply uses the best single SNP p-value within the gene, calculates a gene-based p-value by permutation test within each gene, and assigns that p-value to the gene^27^.

**Supplemental Discussion**

**Accounting for Unknown Covariates Is Critical in Transcriptome Analyses**

In large gene expression studies, variation that confounds results can be introduced at any step despite a tremendous amount of effort to standardize approaches ^28–31^. Fortunately, Surrogate Variable Analysis (SVA) can help to address this by accounting for unknown covariates within large genomic data sets^18^. Importantly, in these analyses, we demonstrate that failure to account for unknown sources of variation leads to an artificially inflated correlation between SCZ and BPD (R=0.50, Supplemental Figure 4). The previously reported correlation between these two disorders (R=0.28) falls between the value reported herein as the correlation between SCZ and BPD (R=0.11, Figure 1 & Supplemental Figure 4) and the correlation reported when unknown covariates fail to be considered (R=0.50). The previously reported correlation between the transcriptomes of SCZ and BPD was likely artificially inflated due to these unknown covariates. We note that the remaining discrepancy between our analysis without SVs included in the linear model and that previously reported is likely due to the fact that our linear model did not include all covariates included in the previous analysis; however, as we did not have access to a number of the technical covariates for the SMRI samples (cDNA concentration, RNA integrity number, or batch number), we were unable to directly test this hypothesis.
