# Supplementary material for: Transcriptome analysis of cortical tissue reveals shared sets of downregulated genes in autism and schizophrenia
Source: Transl Psychiatry. 2016 May 24;6(5):e817–. doi: 10.1038/tp.2016.87 (PMC5070061; doi:10.1038/tp.2016.87)

Supplemental Figure 1

Differential Gene Expression Across AUT, SCZ, and BPD : A density plot for the cross three-disorder Z-scores (Z_{AUT}*Z_{SCZ}*Z_{BPD}) are plotted in black with the cutoff for transcriptome-wide significance highlighted in red ($p<0.05$, determined empirically by permutation). The two genes that meet transcriptome wide significance are labeled.

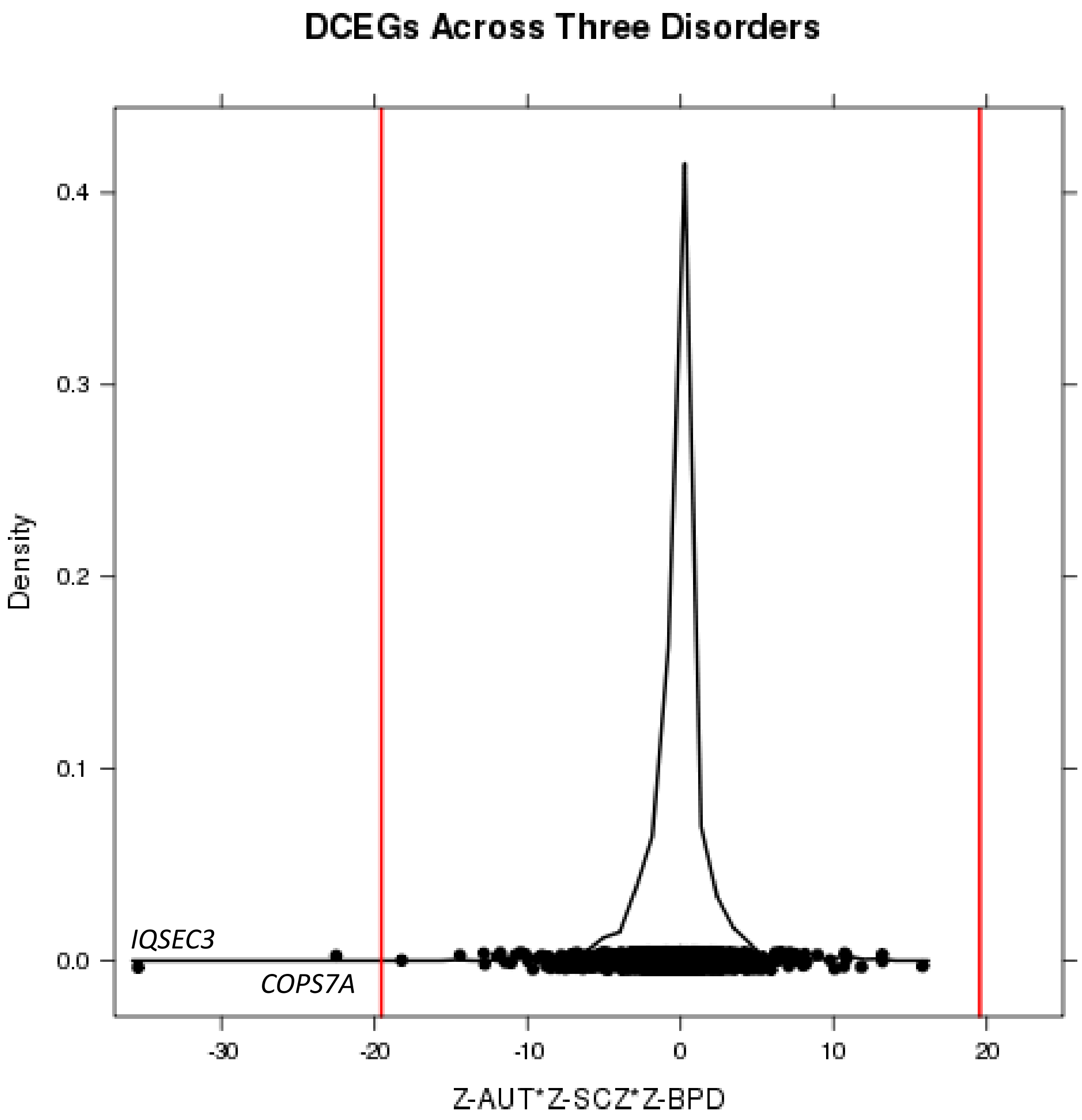

Supplemental Figure 2

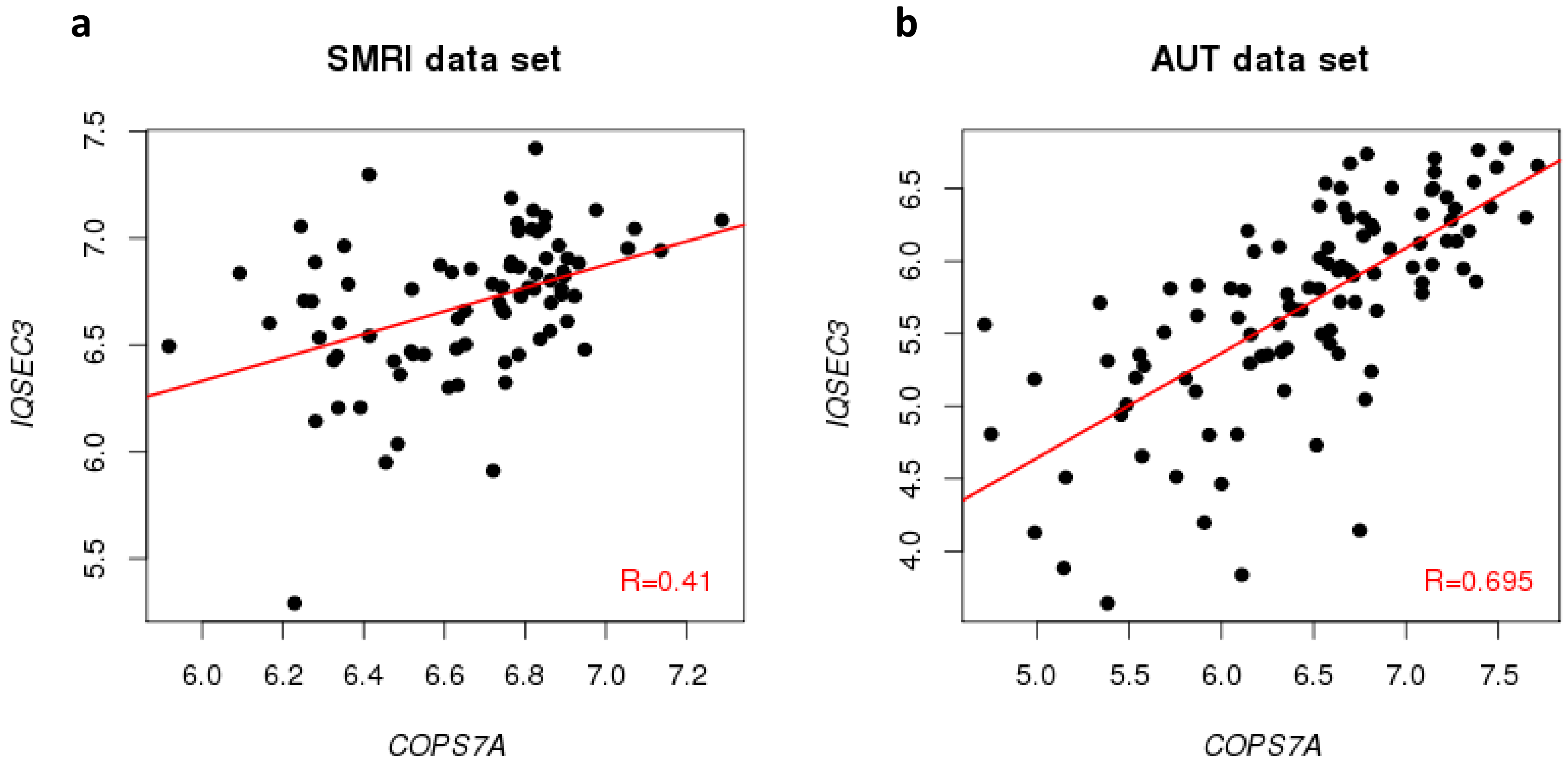

Correlation of Genes Differentially Expressed Across All Three Disorders: The gene expression of *IQSEC* and *COPS7A* in (**a**) the SCZ and BPD data from the SMRI and (**b**) the AUT data are plotted. Pearson's correlation coefficient (R) is in red.

Supplemental Figure 3

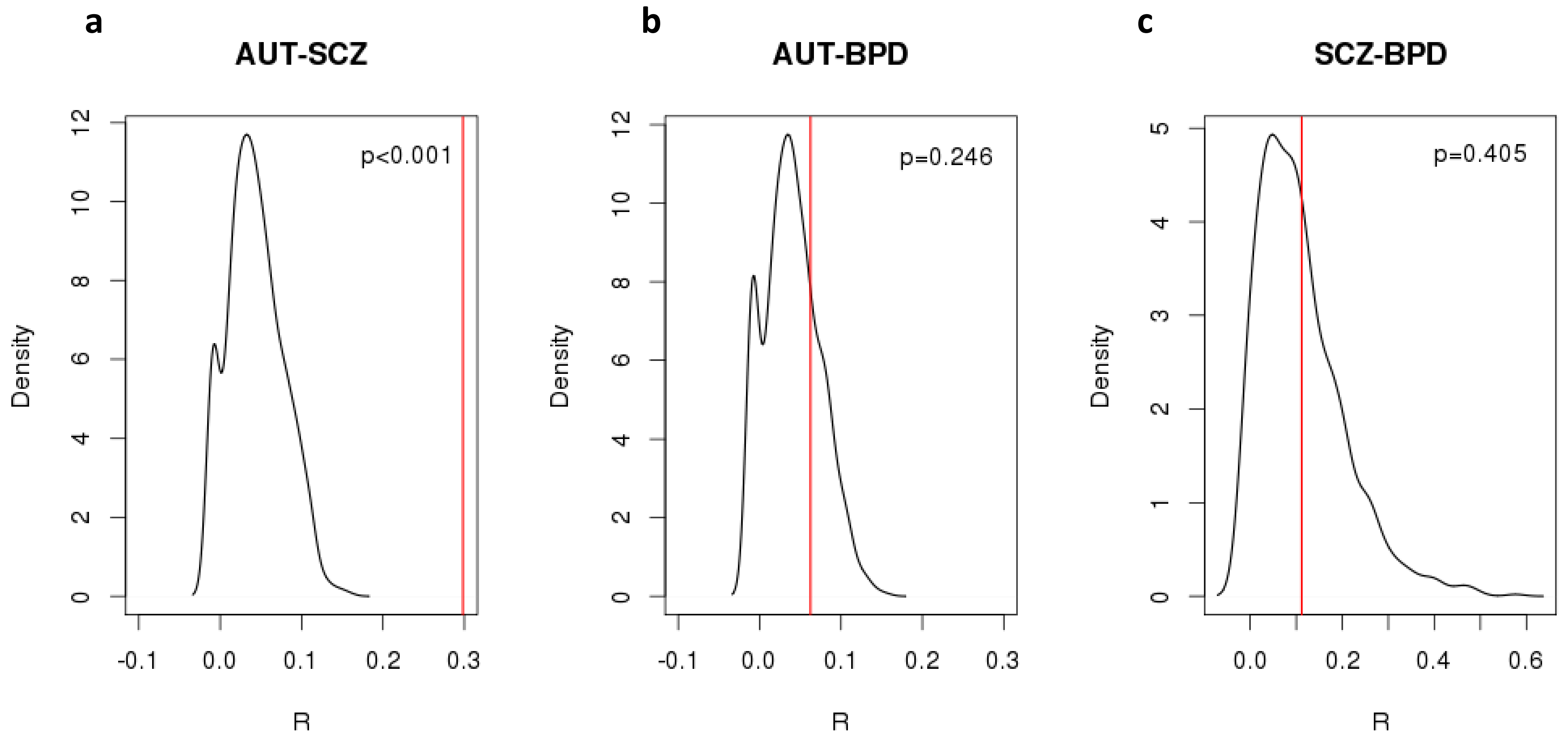

Assessing the significance for Correlations of Cross-Disorder Transcriptomic Similarity: For each cross-disorder comparison, density plots for the correlations of the 1000 null permutations are plotted in black. The cross-disorder correlation derived from the data are plotted in red. **(a)** The correlation between AUT and SCZ is more extreme than the correlation in any of the 1000 null permutations ($p<0.001$). (**b**) The correlation between differential gene expression AUT and BPD is not significant relative to the null correlations ($p=0.246$). (**c**) The correlation between SCZ and BPD is similarly not significant ($p=0.405$).

Supplemental Figure 4

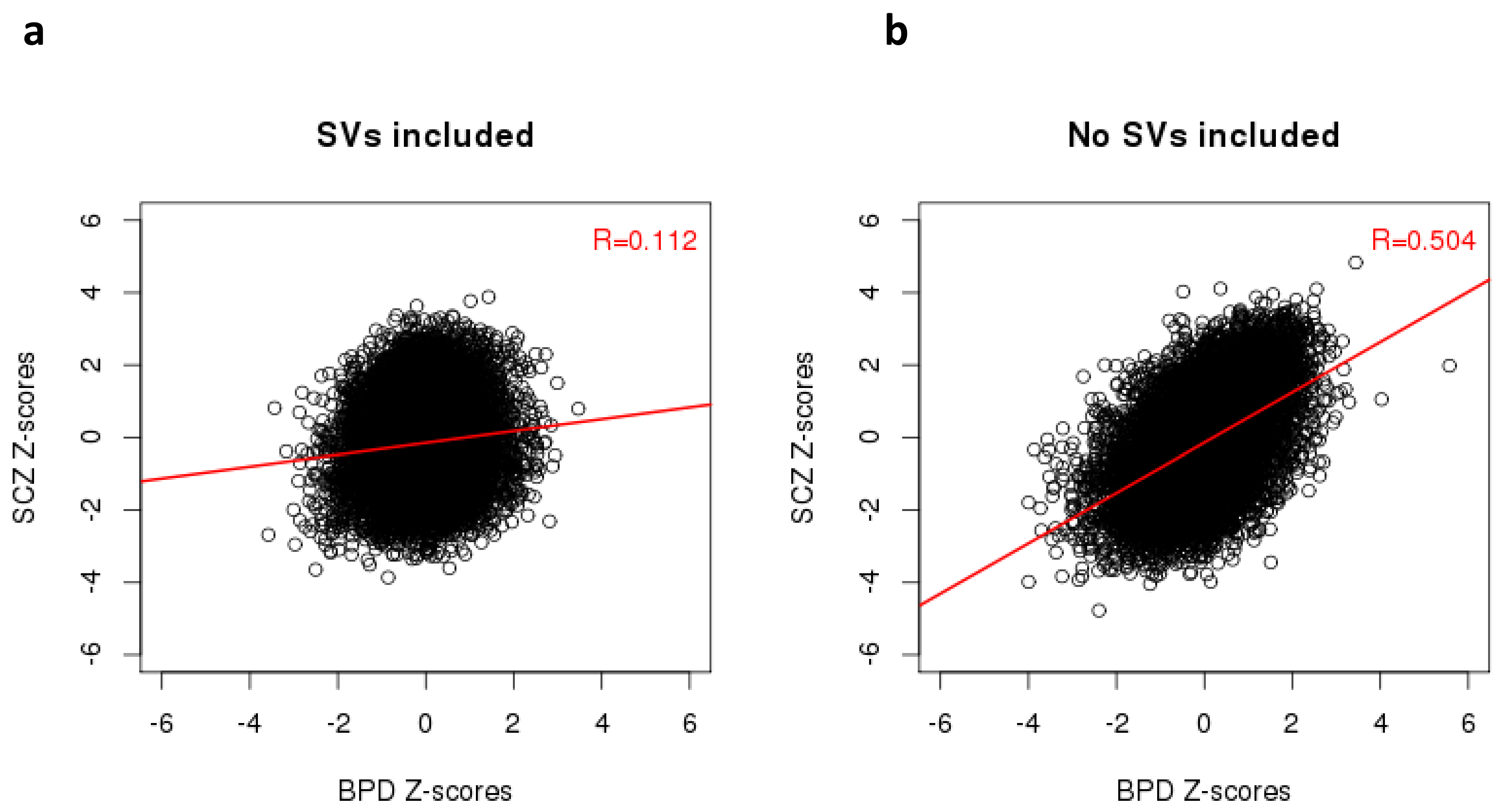

Accounting for Unknown Covariates Affects Correlation: The correlation between differential gene expression in SCZ and BPD reported in this paper in which the linear model included SVs to account for unknown covariates (**a**) relative to an analysis in which these covariates were not included (**b**). The lack of SV inclusion in the linear model to detect differential gene expression leads to an artificially inflated correlation.

Supplemental Figure 5

DCEGs in AUT and SCZ	
Upregulated	Downregulated
NONE	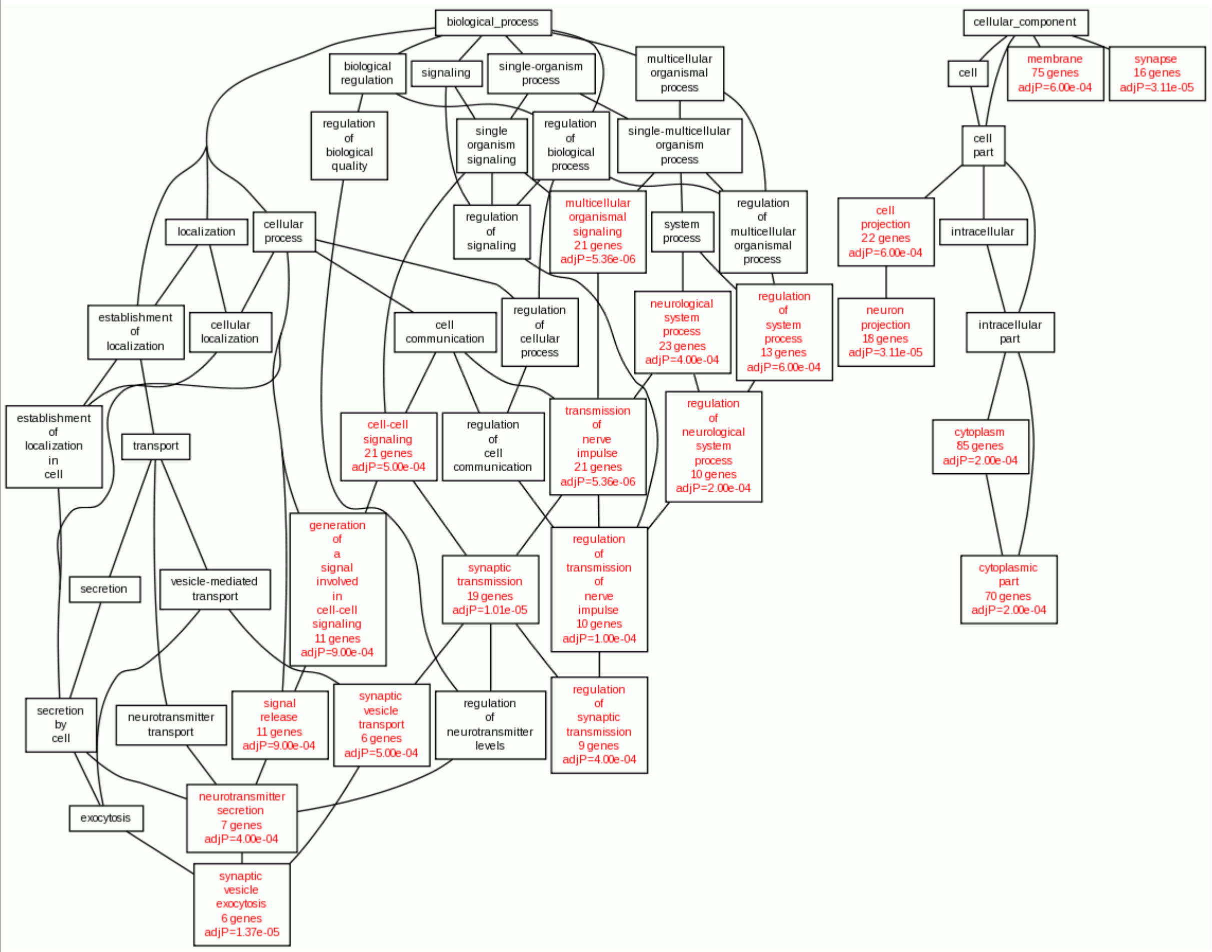

GO Analysis of DCEGs : Genes either concordantly upregulated (left) or concordantly downregulated (right) were analyzed for ontological enrichment of biological processes, developmental processes, and cellular component. Onotological categories with at least five genes and an adjusted p-value < 0.001 are highlighted in red. This abundance of ontological enrichment in those genes concordantly downregulated highlights the role for downregulation of genes differentially expressed in both AUT and SCZ.

Supplemental Figure 6

Cross-Disorder DEGs
(controlling for gene list size)

AUT=SCZ
(191 genes)

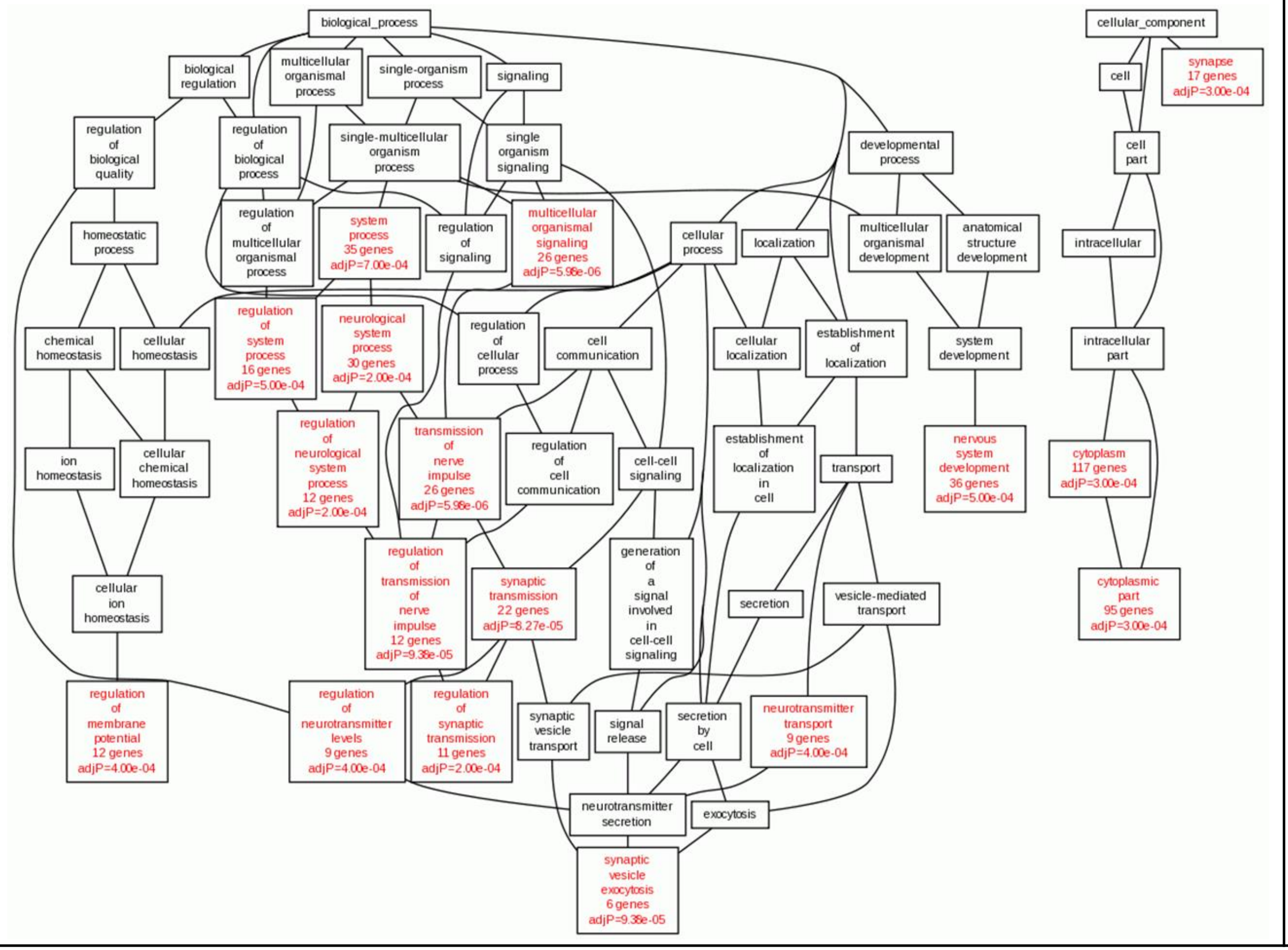

AUT-BPD
(191 genes)

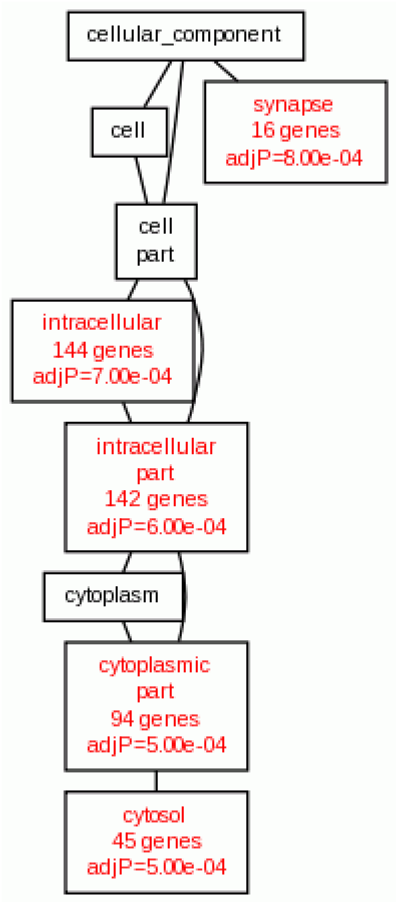

SCZ-BPD
(191 genes)

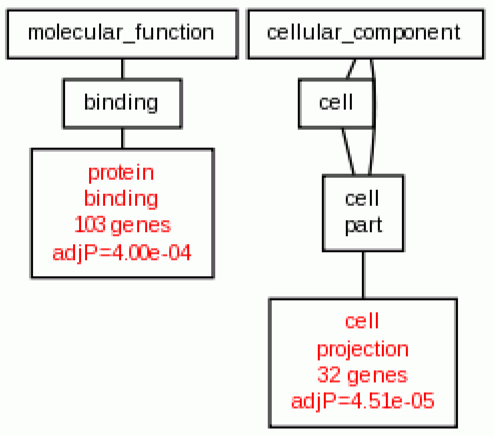

GO Analysis of cross-disorder DEGs : GO results when controlling for the number of genes included in the analysis (rather than using a Z-score cutoff)

Supplemental Figure 7

<table>
<tr><th></th><th>AUT-SCZ</th><th>AUT-BPD</th><th>SCZ-BPD</th></tr>
<tr><td>Downregulated in both</td><td>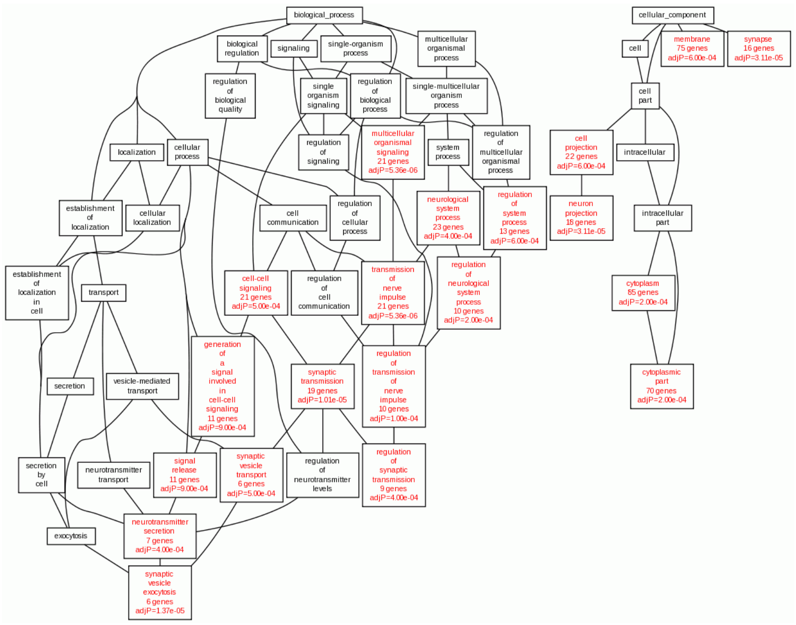
</td><td>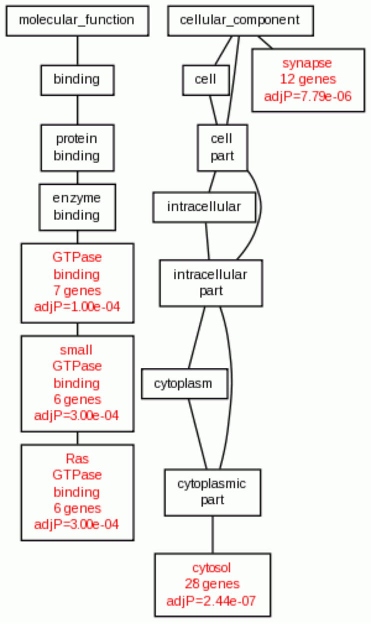
</td><td>NONE</td></tr>
<tr><td>Upregulated in both</td><td>NONE</td><td>NONE</td><td>NONE</td></tr>
</table>

GO Analysis of DCEGs : GO results when both controlling for the number of genes differentially expressed across disorders included in the analysis and only using those genes either downregulated in both disorders (top panel) or upregulated in both (bottom panel) across all three cross-disorder comparisons.

Supplemental Figure 8

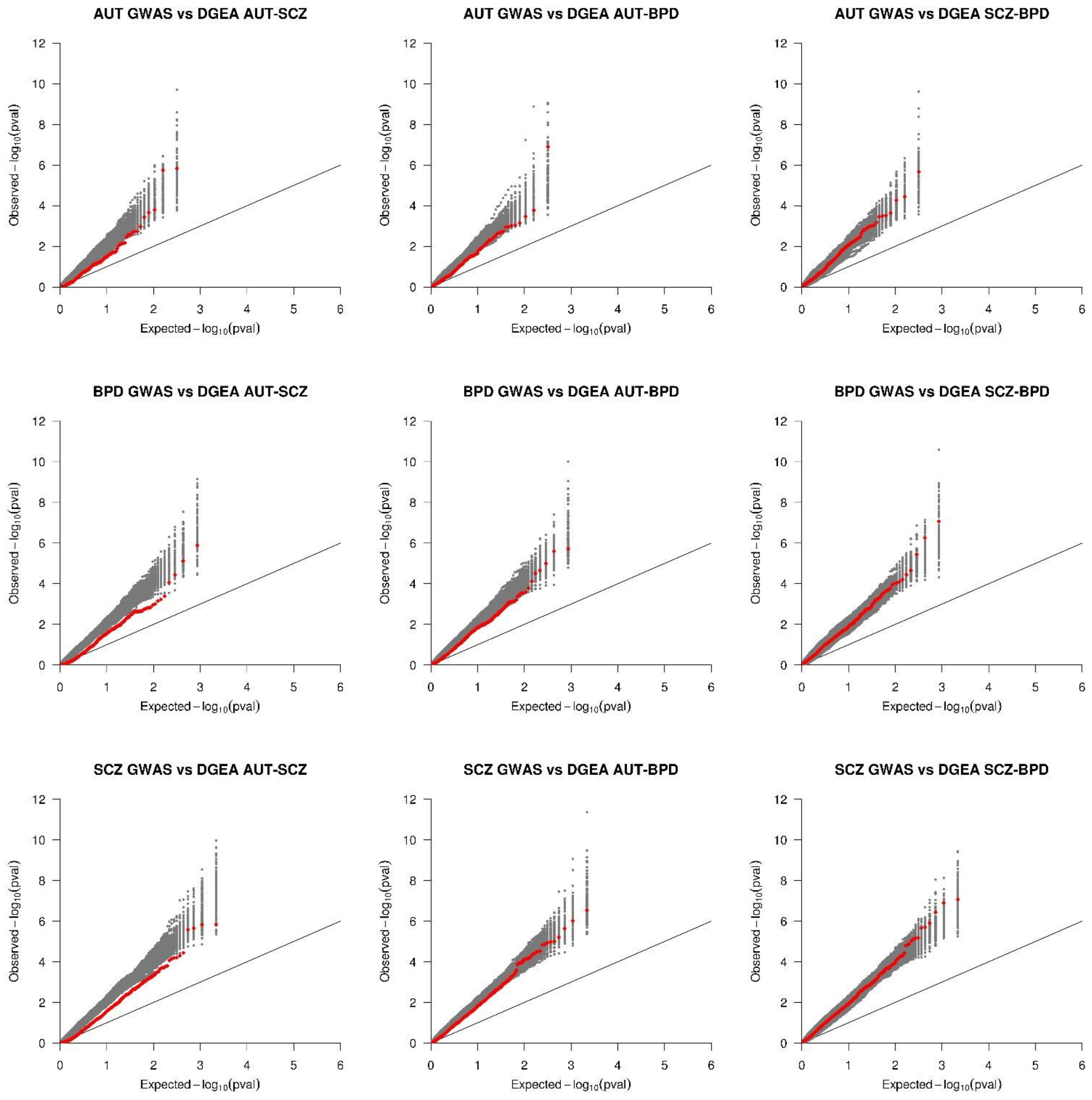

Enrichment of DEGs among GWAS signal : QQ plots assess enrichment of differential gene expression signal (red) among suggestive GWAS results ($p<0.05$). Data for 100 null permutations are plotted in gray. Each row corresponds to GWAS data from a separate disorder (AUT, BPD, SCZ from top to bottom) and each column a different cross-disorder comparison (AUT-SCZ, AUT-BPD, and SCZ-BPD from left to right).

Supplemental Figure 9

Enrichment of DEGs among GWAS at a more permissive p-value cutoff (p<0.1) : QQ plots assess enrichment of differential gene expression signal (red) among suggestive GWAS results (p<0.1). Data for 100 null permutations are plotted in gray. Each row corresponds to GWAS data from a separate disorder (AUT, BPD, SCZ from top to bottom) and each column a different cross-disorder comparison (AUT-SCZ, AUT-BPD, and SCZ-BPD from left to right).

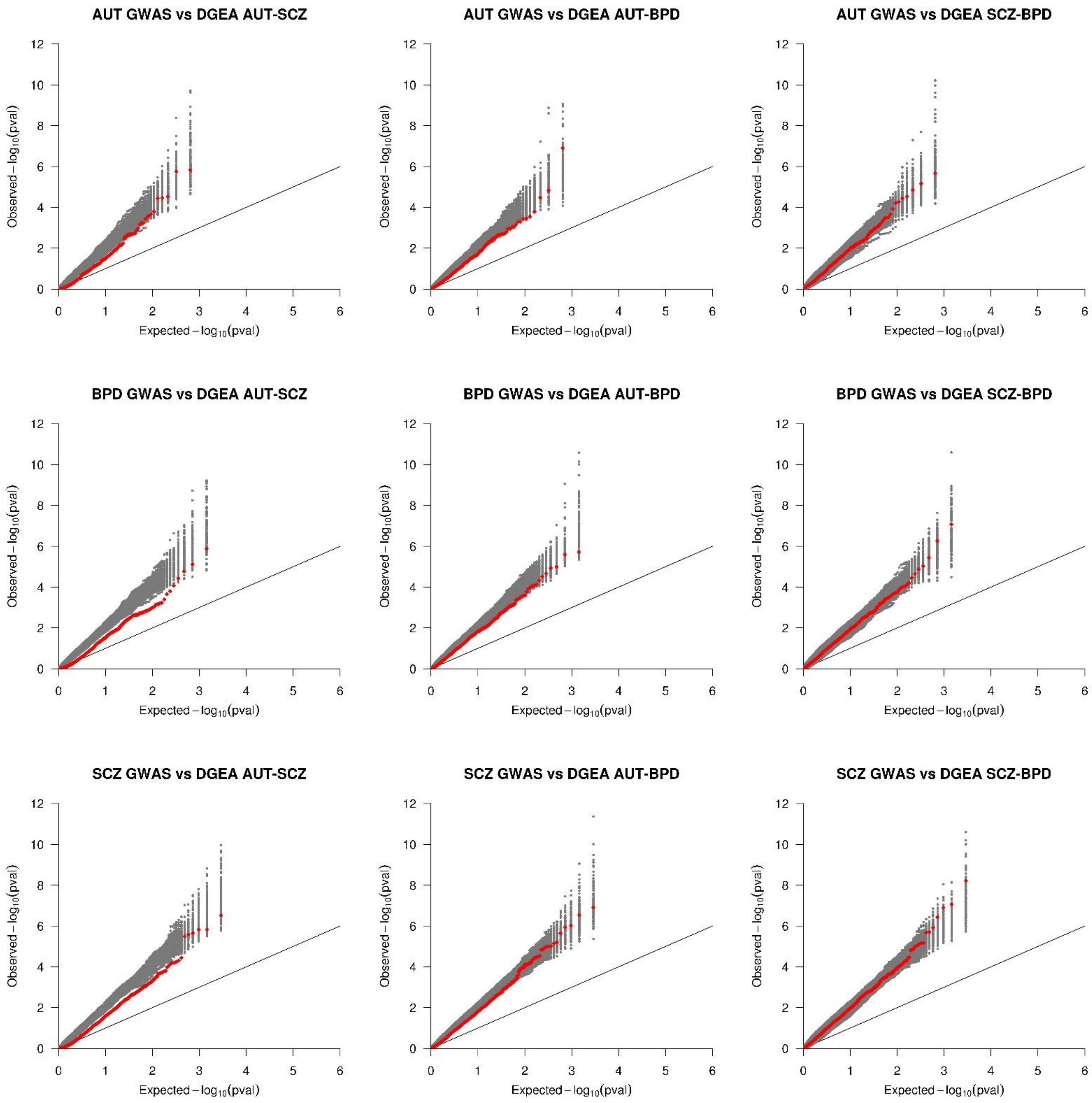

Supplemental Figure 10

Enrichment of DEGs among GWAS signal at a more stringent p-value cutoff (p<0.01) : QQ plots assess enrichment of differential gene expression signal (red) among suggestive GWAS results (p<0.01). Data for 100 null permutations are plotted in gray. Each row corresponds to GWAS data from a separate disorder (AUT, BPD, SCZ from top to bottom) and each column a different cross-disorder comparison (AUT-SCZ, AUT-BPD, and SCZ-BPD from left to right).

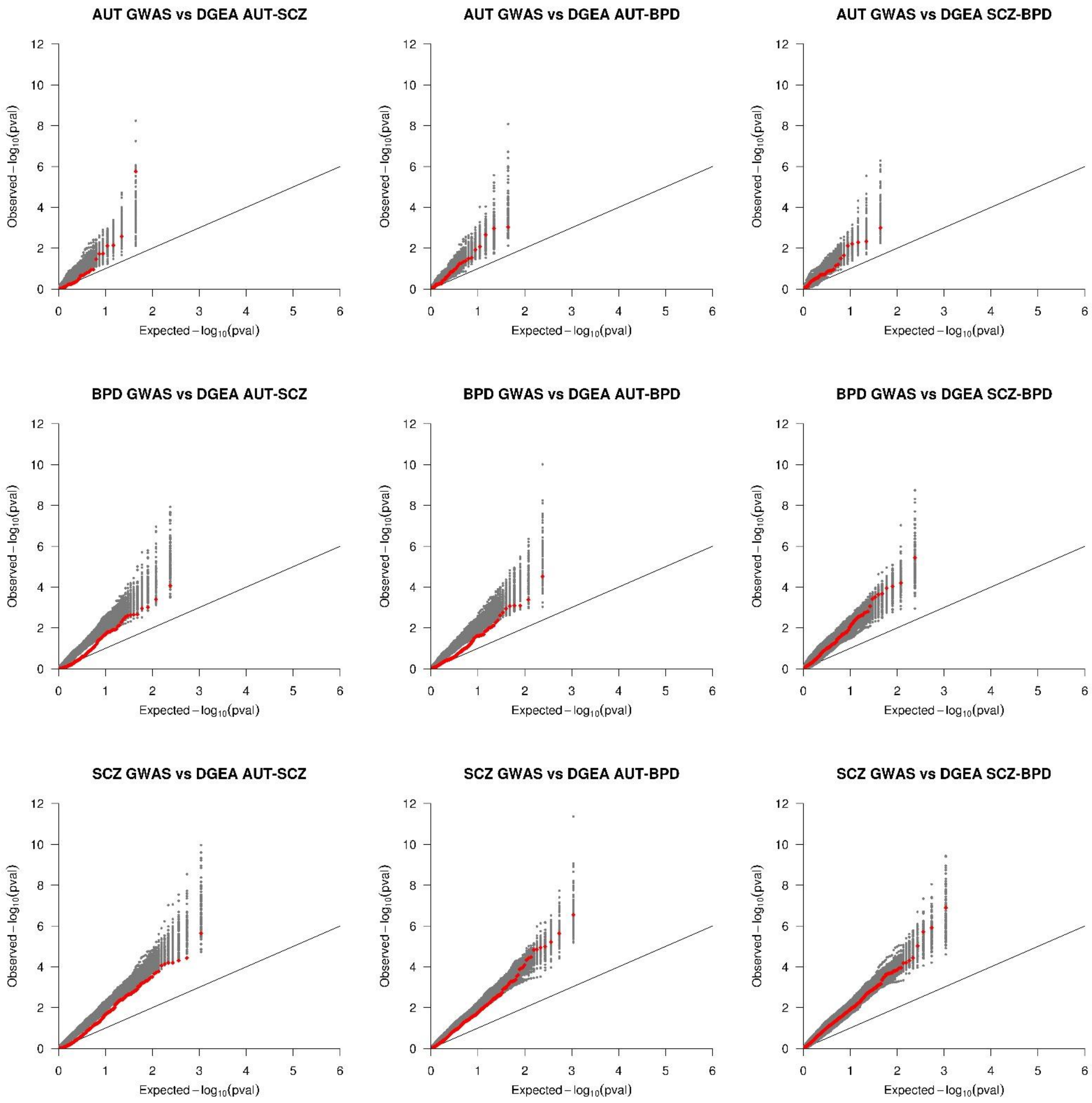

Supplemental Figure 11

Enrichment of DEGs among all genes (no gene based GWAS p-value cutoff imposed) : QQ plots demonstrate that inflation of the test-statistic is present in the data regardless of gene-based p-value cut off. Data for 100 null permutations are plotted in gray. Each row corresponds to GWAS data from a separate disorder (AUT, BPD, SCZ from top to bottom) and each column a different cross-disorder comparison (AUT-SCZ, AUT-BPD, and SCZ-BPD from left to right).

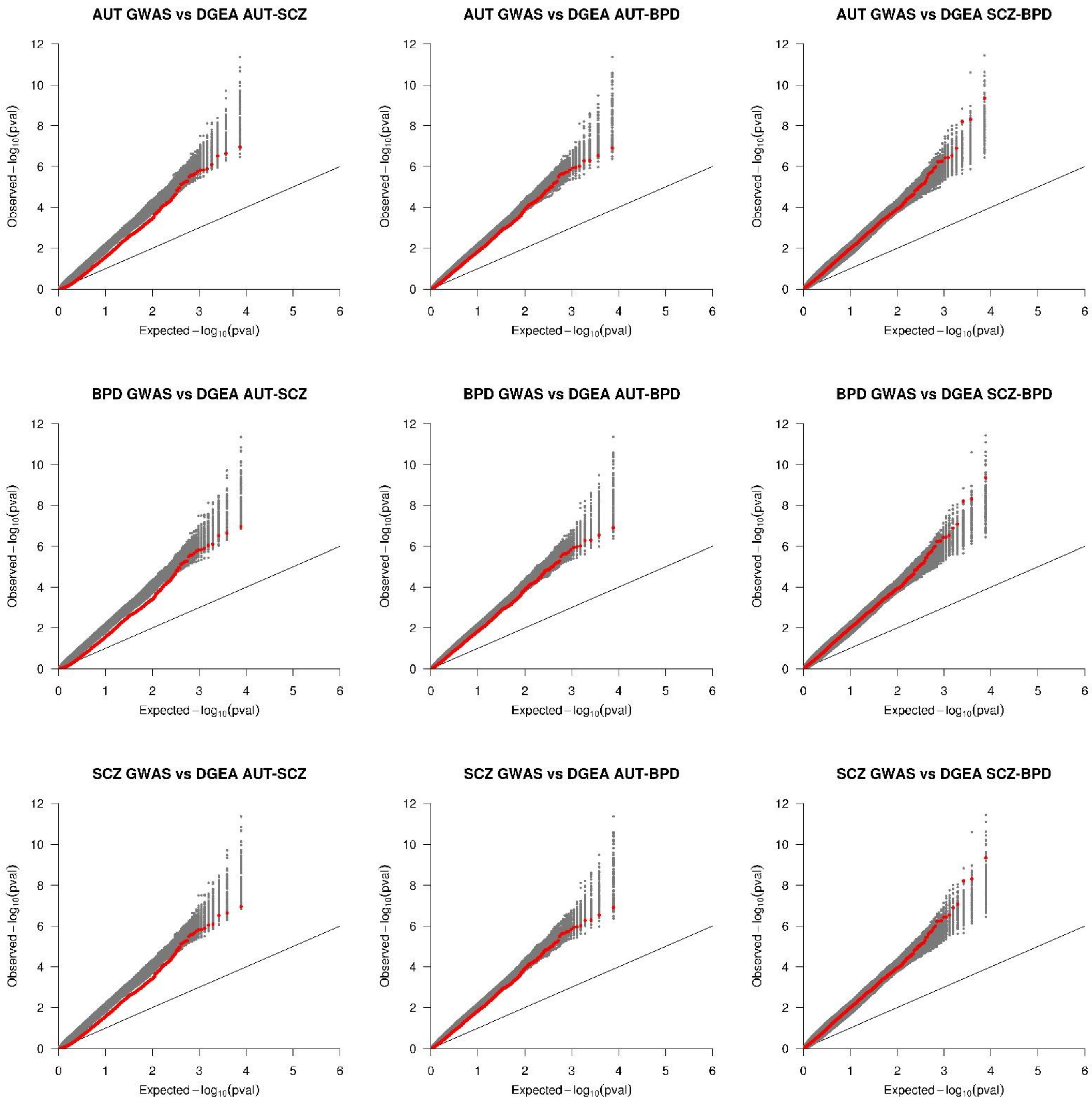

Supplement: Supplementary Figures [file tp201687x3.pdf]
